# Engineered resistance to *Nosema bombycis* by *in vitro* expression of a single-chain antibody in Sf9-III cells

**DOI:** 10.1371/journal.pone.0193065

**Published:** 2018-02-15

**Authors:** Yukang Huang, Jie Chen, Bin Sun, Rong Zheng, Boning Li, Zeng Li, Yaoyao Tan, Junhong Wei, Guoqing Pan, Chunfeng Li, Zeyang Zhou

**Affiliations:** 1 State Key Laboratory of Silkworm Genome Biology, Southwest University, Chongqing, P. R. China; 2 Key Laboratory for Sericulture Functional Genomics and Biotechnology of Agricultural Ministry, Southwest University, Chongqing, P. R. China; 3 College of Life Sciences, Chongqing Normal University, Chongqing, P. R. China; Institute of Plant Physiology and Ecology Shanghai Institutes for Biological Sciences, CHINA

## Abstract

*Nosema bombycis* is a destructive, obligate intracellular parasite of the *Bombyx mori*. In this study, a single-chain variable fragment (scFv) dependent technology is developed for the purpose of inhibiting parasite proliferation in insect cells. The scFv-G4, which we prepared from a mouse G4 monoclonal antibody, can target the *N*. *bombycis* spore wall protein 12 (NbSWP12). Indirect immunofluorescence assays showed that NbSWP12 located mainly on the outside of the *N*. *bombycis* cytoskeleton, although some of it co-localized with β-tubulin in the meront-stage of parasites. When meront division began, NbSWP12 became concentrated at both ends of each meront. Western blotting showed that scFv-G4 could express in Sf9-III cells and recognized native NbSWP12. The transgenic Sf9-III cell line showed better resistance than the controls when challenged with *N*. *bombycis*, indicating that NbSWP12 is a promising target in this parasite and this scFv dependent strategy could be a solution for construction of *N*. *bombycis-*resistant *Bombyx mori*.

## Introduction

Microsporidia are unicellular, intracellular eukaryotic parasites that infect a wide range of eukaryotes including humans, economical fish and insect [1]. *Nosema bombycis*, a silkworm pathogen, is the first microsporidia species identified. It can cause pebrine disease by vertical and horizontal transmission [2, 3]. To some extent, chemical sanitizers such as lime powder, bleaching powder and ClO_2_ can protect silkworms from this disease [4]; however, these methods polluted the environment and significantly increased the production costs of sericulture.

Many microorganisms, such as *Bacillaceae* and yeast, ensure their survival under environmental stress by forming spores. The spore wall of *Saccharomyces cerevisiae* contains four different layers in the form of mannans, glucans, dityrosine cross-linked macromolecules and chitosans [5–7], which is a little different from microsporidia. In contrast, the spore walls of microsporidia contain a proteinaceous, electron-dense exospore, a chitinous, electron-transparent endospore, and a plasma membrane [8, 9]. Microsporidia are equipped with a special organelle, the polar tube, which enables the infection process to occur [10]. When a spore approaches a host cell, it ejects the polar tube to penetrate the cell membrane, injecting the polaroplast into the host cell [11]. After polaroplast entry, the microsporidia begin to proliferate by stealing energy and source materials from the host, which enter the meront life-stage [12]. At this point, the meront has lost the protection of the spore wall and chitin layer.

Spore wall protein 12 (SWP12), a protein located inside and outside of the spore wall, was the first identified Bin/Amphiphusin/Rvs (BAR) domain-containing protein in *N*. *bombycis* [13]. BAR domain proteins play important roles in membrane structure formation, the transport of materials, endocytosis, exocytosis, and signal transduction in yeast [14].

Single-chain antibodies (scFvs) have been used to block transmission of the malaria-causing apicomplexan *Plasmodium*, an obligate intracellular parasite like *N*. *bombycis* [15, 16]. The scFvs, which target two important surface proteins of *Plasmodium falciparum* were expressed in the *Anopheles stephensi* and significantly decreased the infection level of the mosquitoes [17]. As NbSWP12 is also an important surface protein of *N*. *bombycis*, we set out to investigate whether scFvs target to NbSWP12 could inhibit parasite invasion of host cells during the intracellular proliferation stage.

## Materials and methods

### Spore preparation and purification

*N*. *bombycis* CQ1 was obtained from the China Veterinary Culture Collection Center (Accession No. CVCCl02059) [18]. Spores were isolated from infected silkworms and purified by discontinuous Percoll gradient centrifugation (16,000 rpm, 40 min) [19]. Purified spores were stored in ddH_2_O supplemented with antibiotics (Penicillin-Streptomycin solution, 100 ×, Beyotime, China) at 4°C. *Nosema pernyi* was gifted to us by Zhenggang Ma, Chongqing Normal University, China. *Nosema papilio* was prepared and purified for us by Bo Luo (State Key Laboratory of Silkworm Genome Biology, China).

### Protein expression and purification

The vector for expression of the recombinant SWP12 was constructed as previous report [13]. *Escherichia coli* Rosetta, which was used as the bacterial expression strain, was induced for 24 h (16°C, 160 rpm) with 0.5 mM isopropyl-β-D-thiogalactopyranoside in Luria broth. A Ni-nitrilotriacetic acid superflow cartridge was used to purify the recombinant protein according to the manufacturer’s instructions (Roche, Switzerland).

### Spores protein preparation

*N*. *bombycis* proteins were prepared by the glass bead breaking method as described previously [20]. Purified spores, lysed in phosphate-buffered saline (PBS) in the presence of phenylmethylsulfonyl fluoride (Beytime, China), were mixed with 0.4 g of acid-washed glass beads (212–300 μm), and then broken overnight at 4°C using a MT-400 MICRO TUBE MIXER (TOMY Co. Japan). The overnight-treated sample was centrifuged at 12,000 rpm for 5 min and the supernatant was collected. The same method was used to extract proteins from *N*. *pernyi* and *N*. *papilio*.

### Enzyme-linked immunosorbent assay (ELISA)

ELISA plates (96-well, Corning, USA) were coated for 2 h at 37°C with rSWP12 protein (0.4 μg/well) diluted in 0.5 M sodium carbonate buffer, pH 9.5. PBST buffer (137 mM NaCl, 2.7 nM KCl, 10 mM Na_2_HPO_4_, 2 mM KH_2_PO_4_, 0.05% Tween-20, pH 7.4) was then used to wash the plates three times (5 min each time). The plates were blocked for 1 h with 5% skimmed milk, incubated with the test solution (100 μl/well) for 1 h at room temperature, and then washed three times with PBST buffer. Next, 100 μl/well peroxidase-labeled goat anti-mouse IgG (Sigma, Saint Louis, Missouri, USA) (1:3000 in 5% skimmed milk) was added and the plates were incubated for 1 h at room temperature. After washing three times with PBST buffer, 200 μl of TMB Horseradish Peroxidase Color Development Solution for ELISA (Beyotime, China) was added. Controls included culture medium alone or culture medium supplemented with normal or control mouse sera, at dilution ratios consistent with that of the positive control. The reaction was stopped by adding 2 M H_2_SO_4_. The optical density at 450 nm was measured for each well with a microplate reader (TECAN, Switzerland).

### Monoclonal antibody (mAb) production

All animal experiments were conducted in accordance with Laboratory Animals Ethics Review Committee of Southwest University guidelines (Chongqing, China). The committee have approved this study (Permit Number: AERCSWU2017-7). The mice were maintained in accordance with recommendations of the committee for the purpose of control and supervision of experiments on animals by providing food and water ad libitum. MAbs [21] were produced in 6–8 week-old female BALB/c mice. The mice were inoculated subcutaneously by multi-point injection four times with 80–100 μg of rSWP12 mixed with Freund’s complete adjuvant (Sigma, Saint Louis, Missouri, USA) or Freund’s incomplete adjuvant (Sigma, Saint Louis, Missouri, USA) (1:1). One week after final subcutaneous multi-point injections, the mice were intraperitoneally injected with 100 μg of rSWP12 without adjuvant. Four days later, one of the mice was sacrificed by cervical dislocation and sterilized by 75% ethyl alcohol. Spleen cells from the mouse were fused with SP2/0 myeloma cells (10:1) using polyethylene glycol 1500 (Sigma, Saint Louis, Missouri, USA). Fused cells were maintained in RPMI-1640 containing 20% newborn calf serum (SIJIQING Co., China) and hypoxanthine-aminopterin-thymidine (Sigma, Saint Louis, Missouri USA) at 37°C, with 5% CO_2_. Culture supernatants were tested for antibody recognition of the specific antigen by ELISA. Positive hybridomas were sub-cloned three times by limiting dilution. MAb IgG subtypes were identified using a Mouse Monoclonal Antibody Isotyping Kit (Roche, Switzerland) according to the manufacturer’s recommendations.

### Infection of Sf9-III

Newly molted 4th instar silkworm larvae were infected with 1×10^4^ spores per larva by oral feeding. The infections were allowed to propagate in the silkworm larvae until pupation. Then the spores were collected from the blood of the infected silkworm pupae. After priming with 0.1 M KOH, the spores were added to the control Sf9-III cell line which harboring native PiggyBac vector without scFv-G4 coding sequence and to the transgene Sf9-III cell line which harboring modified PiggyBac vector expressing scFv-G4 (spores: cells ratio 1:1) [22]. Infected cells were collected at different time points after infection and then stored in PBS at −80°C.

For transcriptional profile, the Sf9-III cells were also infected by aforementioned method, but the ratio of spores and cells were raised to 10:1. Then the infected cells were collected at 8, 16, 24, 48, 72 and 96 hours post infection. The samples were stored in TRIzol (Invitrogen, Carlsbad, California, USA) at -80°C.

### Indirect immunofluorescent assay (IFA)

Infected cells were fixed in 4% paraformaldehyde, then permeabilized with 0.5% Triton X-100 for 10 min at room temperature, blocked with PBS-bovine serum albumin for 1 h, incubated with mAb (mouse) and polyclonal antibody (rabbit) diluted with PBS-bovine serum albumin at 37°C for 60 min, and finally washed with PBS. Alexa 488 and 594 were used to detect the bound primary antibodies. Chitin was stained with Fluorescent Whitening Agent (FWA) (Sigma, Saint, Louis, Missouri USA) for 20 min. The results were examined by confocal microscopy (Olympus, Japan).

### Gene cloning of mAb

Total RNA was isolated from the G4 hybridoma cell line using the Total RNA Kit II (OMEGA, Norcross Georgia USA). One microgram of RNA was treated with DNase I (TaKaRa, Japan) for each 20 μL reverse transcription reaction. The cDNA of G4 hybridoma cell line was PCR-amplified with the RT-PCR Kit (Promega, Madison, Wisconsin, USA). Heavy-chain and light-chain variable regions were cloned using the following two degenerate primer pairs: VH-F: 5′-AGGTSMARCTGCAGSAGTCWGG-3′, VH-R: 5′- TGAGGAGACGGTGACCGTGGTCCCTTGGCCCC-3′ [23] and VL-F: 5′- GAYATTGTGMTSACMCARWCTMCA-3′ [24], VL-R: 5′- GGATACAGTTGGTGCAGCATC-3′ [25]. The two variable regions were then ligated to pMT19-T for sequence analysis.

### ScFv sequence modifications

According to the results of the aforementioned sequence analysis, five primers were designed for overlapping PCR. The heavy-chain variable region was cloned using HG4 F-Nco I 5′-CATGCCATGGCACCATGCATTGTGAGGTGCAGCTTGTTG-3′ and scV_H_G4 R 5′-AGAGCCGCCACCACCGCTCCCACCACCACCACGGTGACTGAGGTTCCTTGA-3′ primers, meanwhile the light-chain variable region was cloned using scV_L_G4 F5′-AGCGGTGGTGGCGGCTCTGGCGGCGGCGGATCAGACATTGTGATGACCCAGTCT-3′ and scV_L_G4 R 5′-AGAGCCGCCACCACCGCTCCCACCACCACCTGAGGAGACGGTGACTGAGGT-3′ primers. The two variable regions were connected by overlapping PCR using HG4 F-NcoI and scV_L_G4 R1-NotI 5′-TTAGCGGCCGCTCATTACTTATCGTCGTCATCCTTGTAATCTGACCCTG-3′ primers, and a short polypeptide linker (G_4_S)_3_ was inserted between the two variable regions at this step. A FLAG-tag was also added to the end of the scFv sequence via a G_3_S_2_ linkage. The above overlapping PCR products were ligated to pMT19-T (TaKaRa, Japan), and the resulting plasmid was digested with Not I and Bam HI restriction enzymes. The enzyme-digested product was ligated to the pSL-1180 plasmid which contain IE1 promoter, multiple cloning sites and SV40 to generate pSL[IE1-scFvG4], then the IE1-scFv-G4-SV40 expression cassette was obtained via enzyme digestion of pSL[IE1-scFvG4] with Bgl II. Finally, the expression cassette was inserted into a PiggyBac vector containing the enhanced green fluorescent protein gene (EGFP) and the neomycin resistance gene (*neo*) to generate PiggyBac[A3-EGFP+A3-Neo+IE1-scFvG4].

### Construction of scFv-G4 transgenic Sf9-III

The PiggyBac[A3-EGFP+A3-Neo+IE1-scFvG4] was extracted from the DH5α cells with the EndoFree Mini Plasmid Kit II (TIANGEN, China). Sf9-III cells were transfected with 2 μg of this plasmid and a helper plasmid using X-tremeGENE HP DNA Transfection Reagent (Roche, Switzerland), and the culture medium was changed after 6 h. Three days later, the cells were cultured in Sf-900 III SFM (Thermo Fisher Scientific, Waltham Mass USA) containing G418 (200 μg/mL) (Merck, Germany), and the culture medium was changed once every 3 d. The screening lasted for two months [26, 27].

### Real-time quantitative PCR analysis

The RNA samples of transcript profile were extracted using a Total RNA Kit II (OMEGA, Norcross, Georgia, USA). 1 μg of each RNA samples were reverse transcribed into cDNA by EvoScript Universal cDNA Master Kit (Roche, Switzerland). The Real-time quantitative PCR were conducted using NbSWP12-qF2 5’-AACATGGATAAAGTAGCCACAGG-3’, NbSWP12-qR2 5’-TGCGAGCAAATCCTTCAAAA-3’ primers and reference gene primers SSU-qF 5’-CTGGGGATAGTATGATCGCAAGA-3’, SSU-qR 5’- CACAGCATCCATTGGAAACG-3’. Real-time PCRs were performed with an initial denaturation of 95°C for 2 min, followed by 40 cycles at 95°C for 10 s and 60°C for 20 s (CFX96^TM^ Real-Time System, Bio-Rad, Richmond, California, USA).

Infected cells collected at 1, 3, 5, 7 days post-infection (d.p.i), were subject to genomic DNA (gDNA) extraction using a DNA extraction Kit (OMEGA, Norcross, Georgia, USA). The gDNA samples were diluted 10-fold, and 1 μL of each diluted sample was used for Real-time quantitative PCR. The 10 μL reaction mixtures were amplified using *β-Nbtubulin*-qF 5′-AGAACCAGGAACAATGGACG-3′ and *β-Nbtubulin*-qR 5′-AGCCCAATTATTACCAGCACC-3′ primers. The resultant amplicons were cloned into pMD19-T and then used as the standards. Real-time quantitative PCRs were also performed with above method. The standard curve covered four orders of magnitude for the copy numbers (1.3×10^3^–10^6^).

### Immunoblot analysis

Spore proteins, rSWP12 and transgenic cells total protein were separated by sodium dodecyl sulfate polyacrylamide gel electrophoresis (SDS-PAGE), and then transferred to polyvinylidene fluoride (PVDF) membranes (Roche, Switzerland). Each membrane was blocked in 5% skimmed milk diluted in TBST (20mM Tris-HCl, 150mM NaCl, 0.05% Tween-20), incubated with mAb G4 or an anti-FLAG mouse antibody (diluted 1:3000; Sigma, Saint Louis, USA) and HRP-labeled goat anti-mouse IgG (diluted 1:6000; Bio-Rad, Richmond, California, USA), successively, with washing in between. ECL Plus Western Blotting Detection Reagents (Bio-Rad, Richmond, California, USA) were used to detect the bound antibodies. ScFv specificity was determined by immunoblots through using the scFv-G4 as primary antibody, which was extracted from the scFv-G4 expressing Sf9-III cells. The rest of the steps were same as the aforementioned protocol.

## Results

### Monoclonal antibody against SWP12

**Fig 1:**
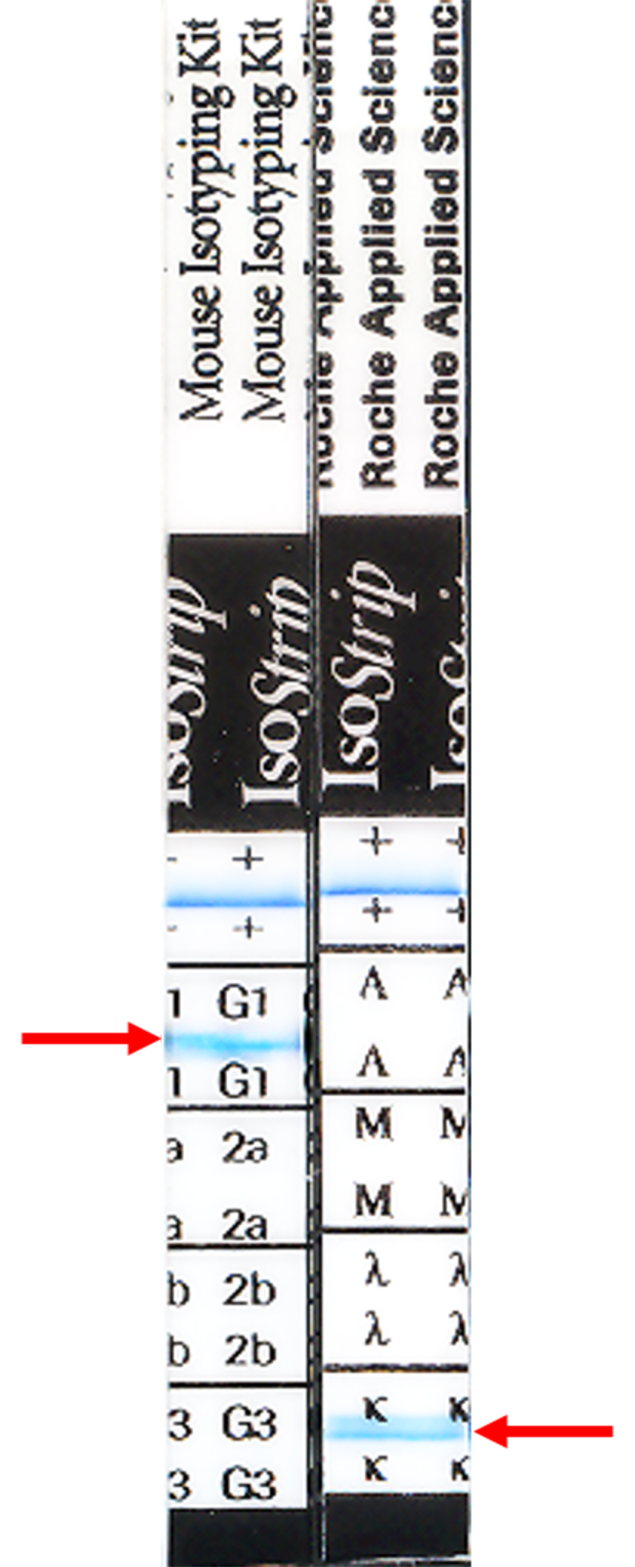
Subtypes identification of G4. The left red arrow indicated the subtype of heavy-chain was IgG1, while the right arrow showed the light-chain subtype was kappa (κ). The blue line in the ‘+’ region is control line.

**Fig 2:**
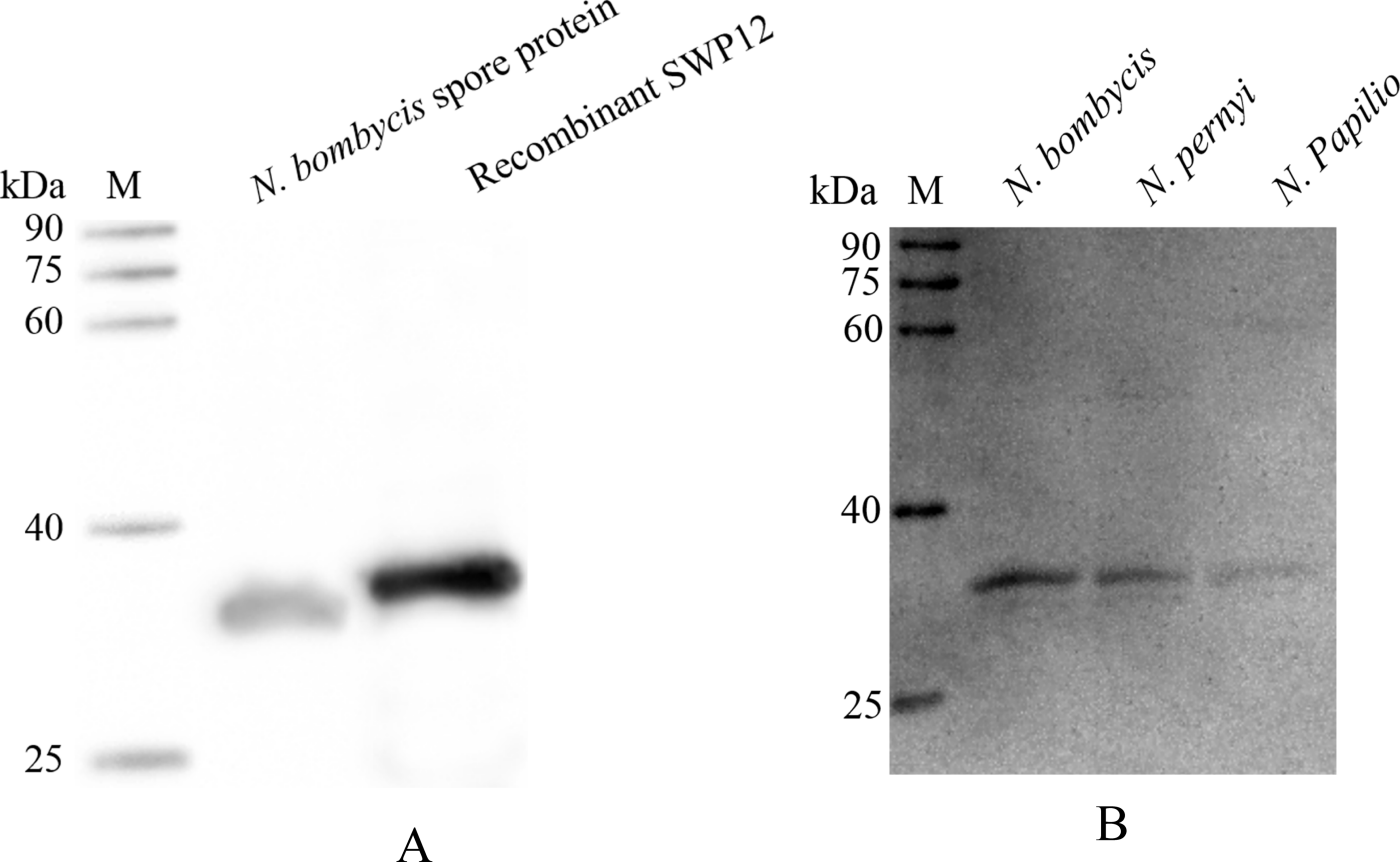
Specificity of the G4 mAb. (A) The protein of *Escherichia coli* Rosetta which was constructed for expression of rSWP12 and *N*. *bombycis* spore protein were used to detect specificity of G4 mAb by western blotting. (B) Spore proteins extracted individually from *N*. *bombycis*, *N*. *pernyi* and *N*. *papilio* were also subjected to SDS-PAGE and western blotting using the G4 mAb against SWP12. M: protein marker (Transgene, China).

Purified rSWP12 (S1 Fig) was used as an antigen to inoculate BALB/c mice and thereafter screen the hybridomas. The splenocytes of one of the above-mentioned BALB/c mice were separated and fused with SP2/0 by mediation of polyethylene glycol 1500. After three rounds of screening, the G4 mAb showed a marked positive reaction against rSWP12 in ELISAs. The titer of G4 hybridoma medium and G4 ascites were measured by ELISA (S2A and S2B Fig), which were 1:2048 and 1:51200 respectively. And the G4 hybridoma serum-free medium was used to detect the subtypes of G4. The test strip proved that the subtypes of G4 were IgG1-κ (Fig 1). Western blotting was conducted to determine whether the G4 mAb could recognize rSWP12 and native SWP12 in spore protein extracted individually from *N*. *bombycis*, *N*. *pernyi* and *N*. *papilio*. The mAb G4 generated specific signals in rSWP12 and each spore protein sample from different microsporidians (Fig 2A and 2B). The tag protein fused in rSWP12 led to the molecular weight difference between rSWP12 and native SWP12 in spore protein (Fig 2A). Western blotting and ELISA implied the high specificity and affinity of the mAb against SWP12, which laid the foundation for neutralizing experiment.

### Transcript profile of *SWP12* in infected Sf9-III cells

**Fig 3:**
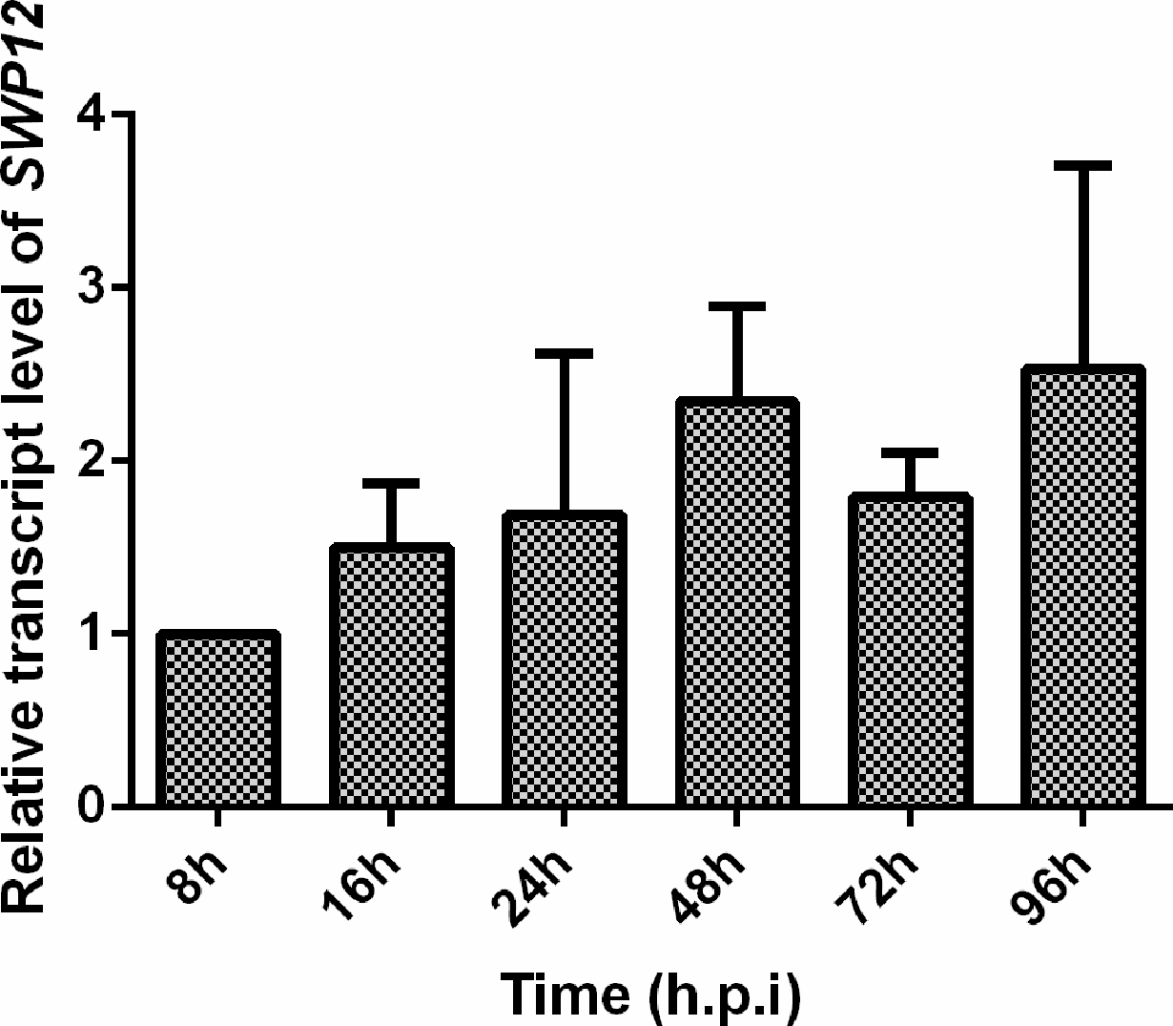
Transcript profile of *SWP12* in infected Sf9-III cells. The relative expression level of *NbSWP12* in the Sf9-III at different time points after infection with *N*. *bombycis*, which was presented relative to 8 h.p.i sample that was arbitrarily assigned a value of 1. The y-axis indicates the relative expression level of *NbSWP12*, and the x-axis indicates the time post infection. *N*. *bombycis* SSU rRNA was reference gene for normalization of expression level. Vertical bars represent the mean ± SE (n = 3).

After infecting Sf9-III cells with *N*. *bombycis*, *SWP12* began to express at early stage of infection and increased gradually at 16 to 48 h.p.i. Then expression level maintained relatively stable at 48 to 96 h.p.i (Fig 3). Spore wall proteins of microsporidia provide protection and resistance against adversity in mature stage [28]. According to the transcript profile, *SWP12* not only expressed at the late stage of infection which was related to mature spore formation, but also transcribed during meront stage.

### Subcellular localization of SWP12 in *N*. *bombycis* meronts

**Fig 4:**
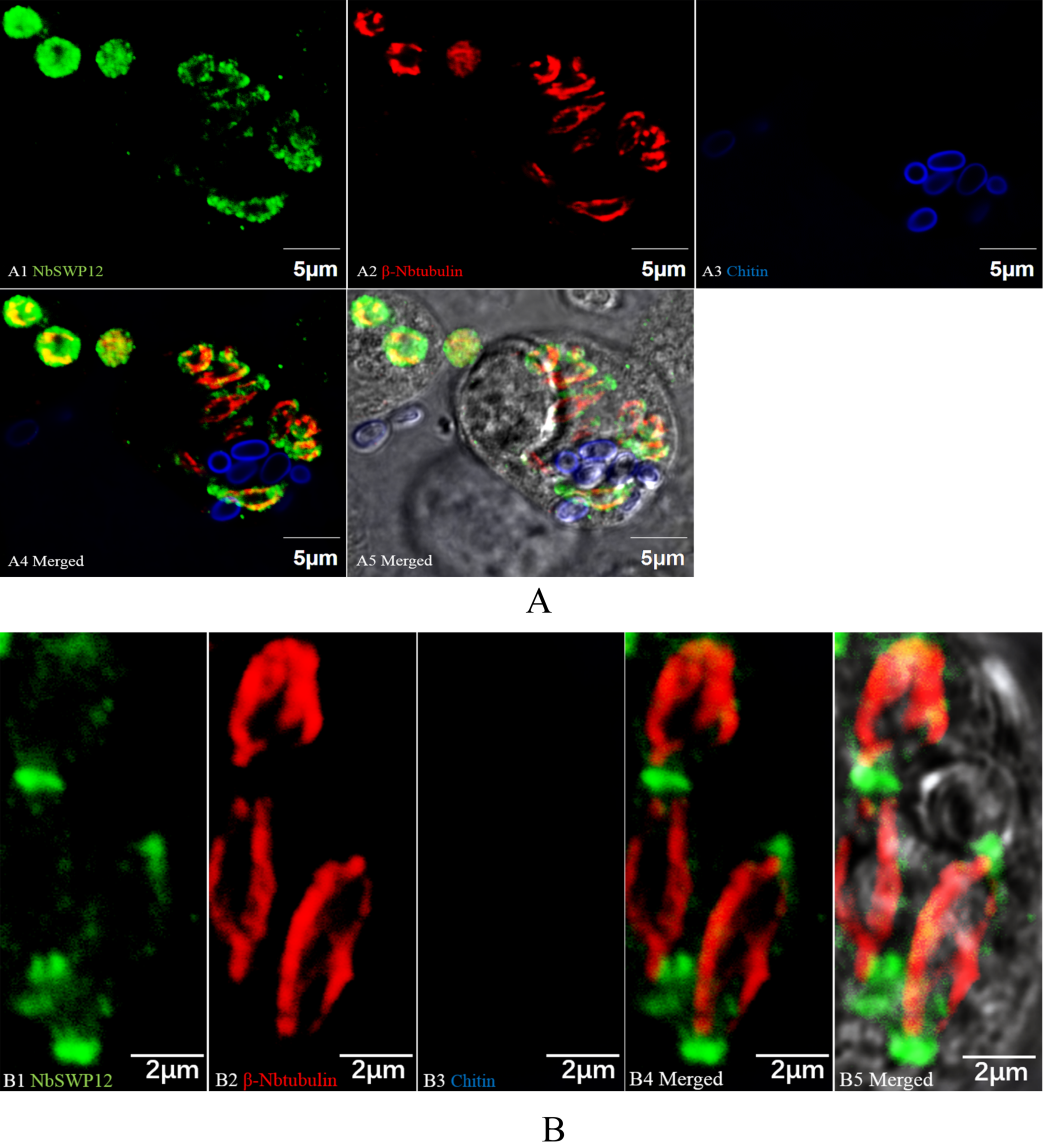
Subcellular localization of SWP12 in meront-stage *N*. *bombycis*. Sf9-III cells were infected with *N*. *bombycis* and the resultant infections were visualized by confocal microscopy. Green and red fluorescence was observed in the samples treated with the G4 mAb against SWP12 and rabbit β-tubulin antiserum, respectively. The blue fluorescent signal represents the chitin coat labeled with Fluorescent Whitening Agent (FWA) (Sigma, Saint Louis, Missouri, USA). (A) Localization of SWP12 in different life stages of *N*. *bombycis*. Meronts and mature spores are distinguishable by β-tubulin antiserum and FWA, respectively. (B) Detailed illustration of SWP12 localization during meronts division. The SWP12 signal concentrated at both ends of the meronts.

As a member of the BAR family, SWP12 may play an important role in meronts. Mature spores contain a thick chitin coat and a large number of spore-wall proteins on the outside [28], and these form a barrier resistant to antibodies bond; however, the meront stage is just enveloped by a cell membrane making it more susceptible to antibody targeting. Therefore, *N*. *bombycis* β-tubulin antibody and chitin dye (FWA)(Sigma, Saint Louis, Missouri, USA) were used as markers to distinguish mature spores and the meront [29]. Infected Sf9-III cells were used for IFAs three days after infection. The IFAs showed that SWP12 located outside of the *N*. *bombycis* cytoskeleton, while part of it also co-localized with β-tubulin (Fig 4A). When the meronts began to divide, the signal from SWP12 tended to be concentrated at both ends of the meronts (Fig 4B). Subcellular localization of SWP12 in *N*. *bombycis* meronts implied SWP12 was not only a spore wall protein which could protect the spore from adversity, but also a potential membrane protein functioning during cell division.

### Construction of scFv-G4

**Fig 5:**
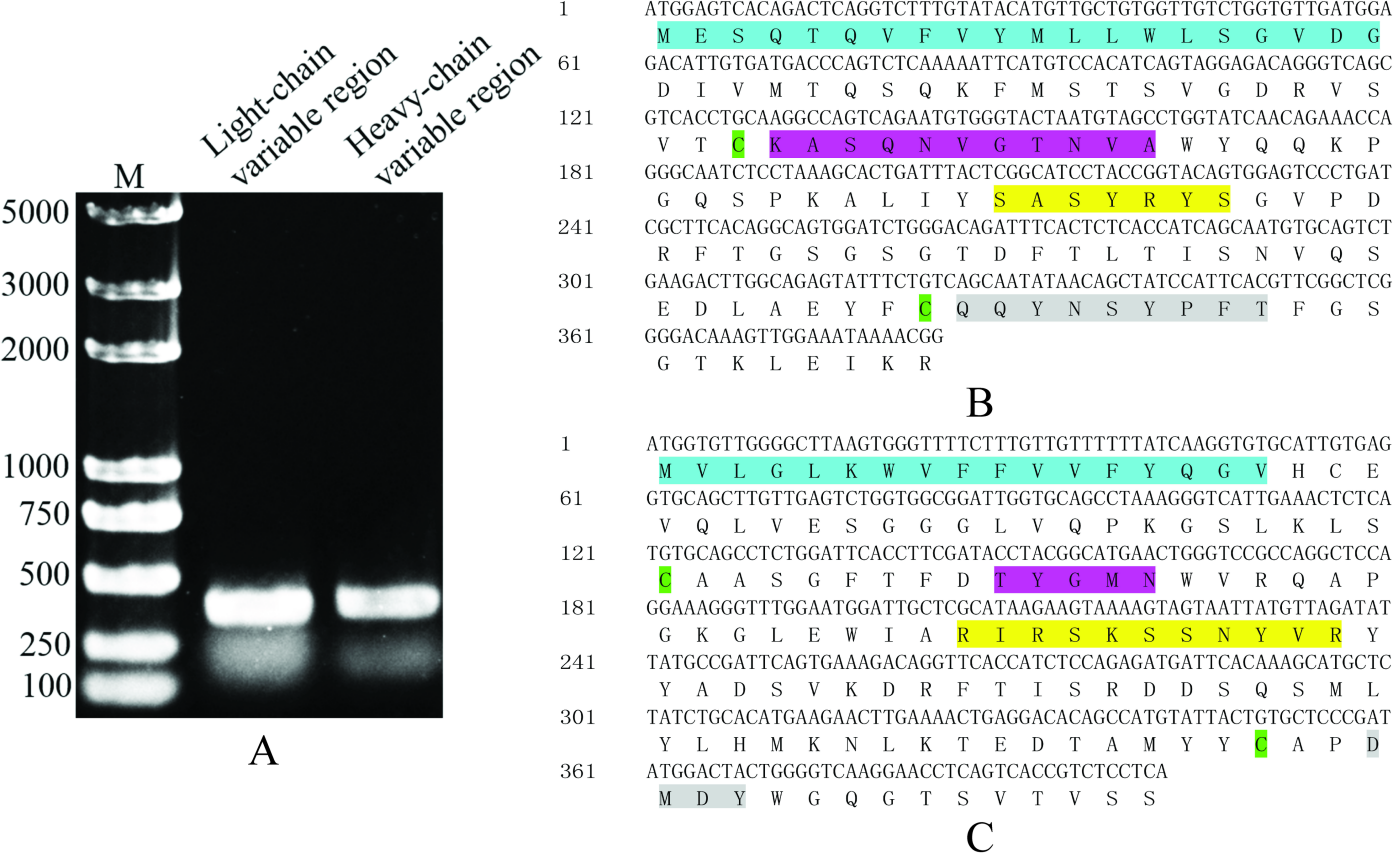
The clone of heavy-chain, light-chain variable regions. (A) Heavy-chain, light-chain variable regions were cloned from G4 hybridoma with degenerate primers by RT-PCR. The two amplicons were sequenced respectively. (B) Light-chain variable region sequence (Accession numbers: MF459004). (C) Heavy-chain variable region sequence (Accession numbers: MF459005). Blue: signal peptide; Green: cysteine; Pink: complementarity-determining region (CDR)1; Yellow: CDR2; Gray: CDR3.

**Fig 6:**
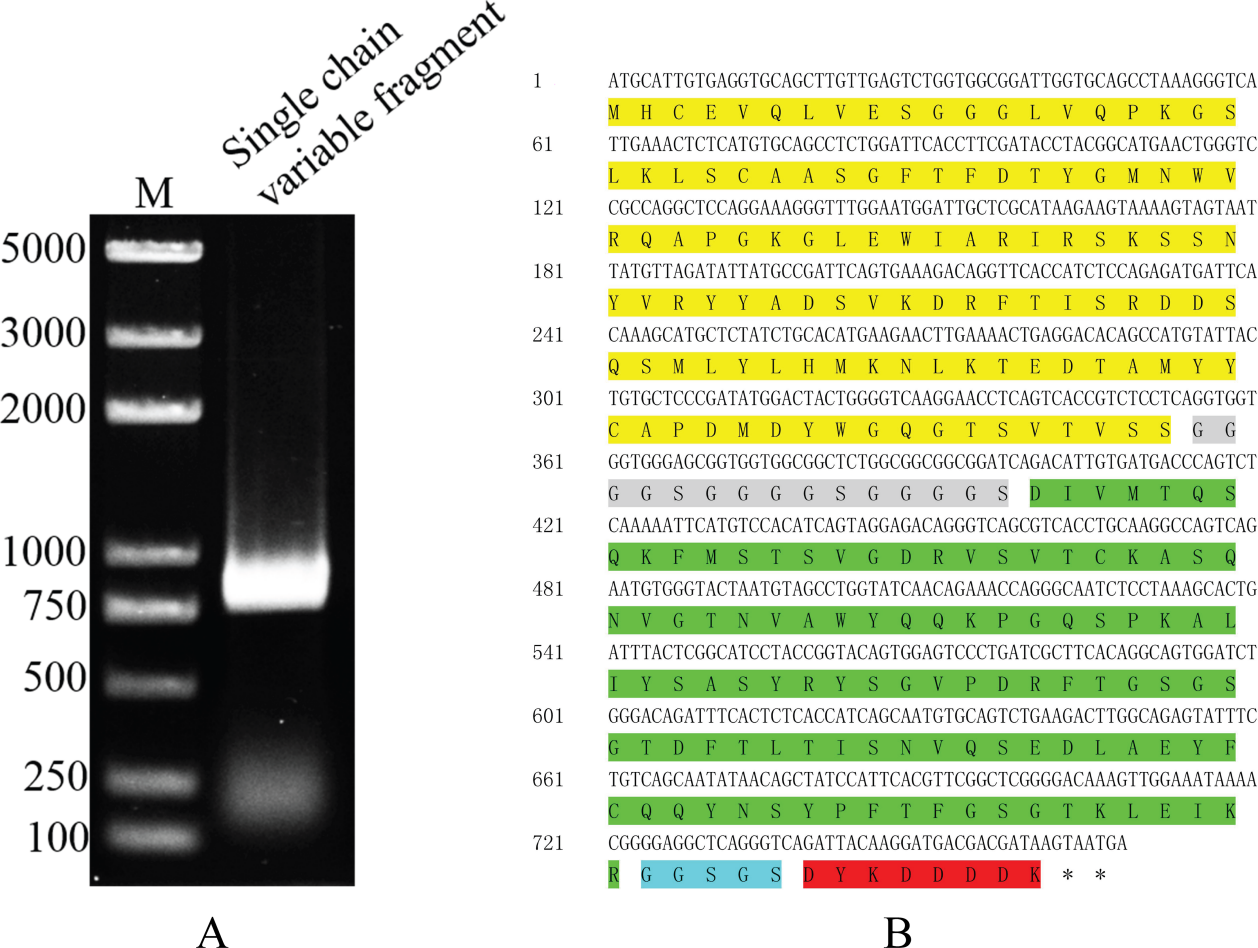
The construction of scFv-G4. (A) Overlap PCR was used to link the two variable region sequences together. (B) Analysis of constructed scFv sequence (Accession numbers: MF459006). Yellow: light-chain variable region sequence; Gray: (G_4_S)_3_ linker; Green: heavy-chain variable region sequence; Blue: G_3_S_2_linker; Red: FLAG-tag.

The G4 light-chain and heavy-chain variable region coding sequences were amplified from the G4 hybridoma cDNA by PCR with degenerate primers which was chose on the basis of subtypes of G4 (Fig 5A). Then the two amplicons were inserted into pMD19-T vector respectively and sequenced with M13-F/R primers. The sequence results of light-chain and heavy-chain variable regions were submitted to NCBI GenBank (Accession numbers: MF459004, MF459005 respectively). The sequencing results were analyzed by IgBLAST (https://www.ncbi.nlm.nih.gov/igblast/). The heavy-chain and light-chain variable regions included the signal peptides, three complementarity-determining regions and two disulfide-bond forming cysteines (Fig 5B and 5C). The analysis result showed the integrated variable region of G4 was cloned with degenerate primers. Based on the sequencing results, the primers of the next overlap PCR were designed. The overlap PCR was conducted to link light-chain and heavy-chain together (Fig 6A). Because SWP12 functions in the intracellular meront stage of *N*. *bombycis*, the signal peptides were deleted in the constructed scFv. Heavy-chain and light-chain variable regions were linked via (G_4_S)_3_ linkers, and FLAG-tag was added to the C-terminus by a G_3_S_2_ linker (Fig 6B). The sequence of constructed scFv was also submitted to GenBank (Accession numbers: MF459006). The constructed scFv-G4 was inserted into pSL-1180 and PiggyBac successively to generate pSL[IE1-scFvG4] (S3A Fig) and PiggyBac[A3-EGFP+A3-Neo+IE1-scFvG4] (S3B Fig) for expression in Sf9-III.

**Fig 7:**
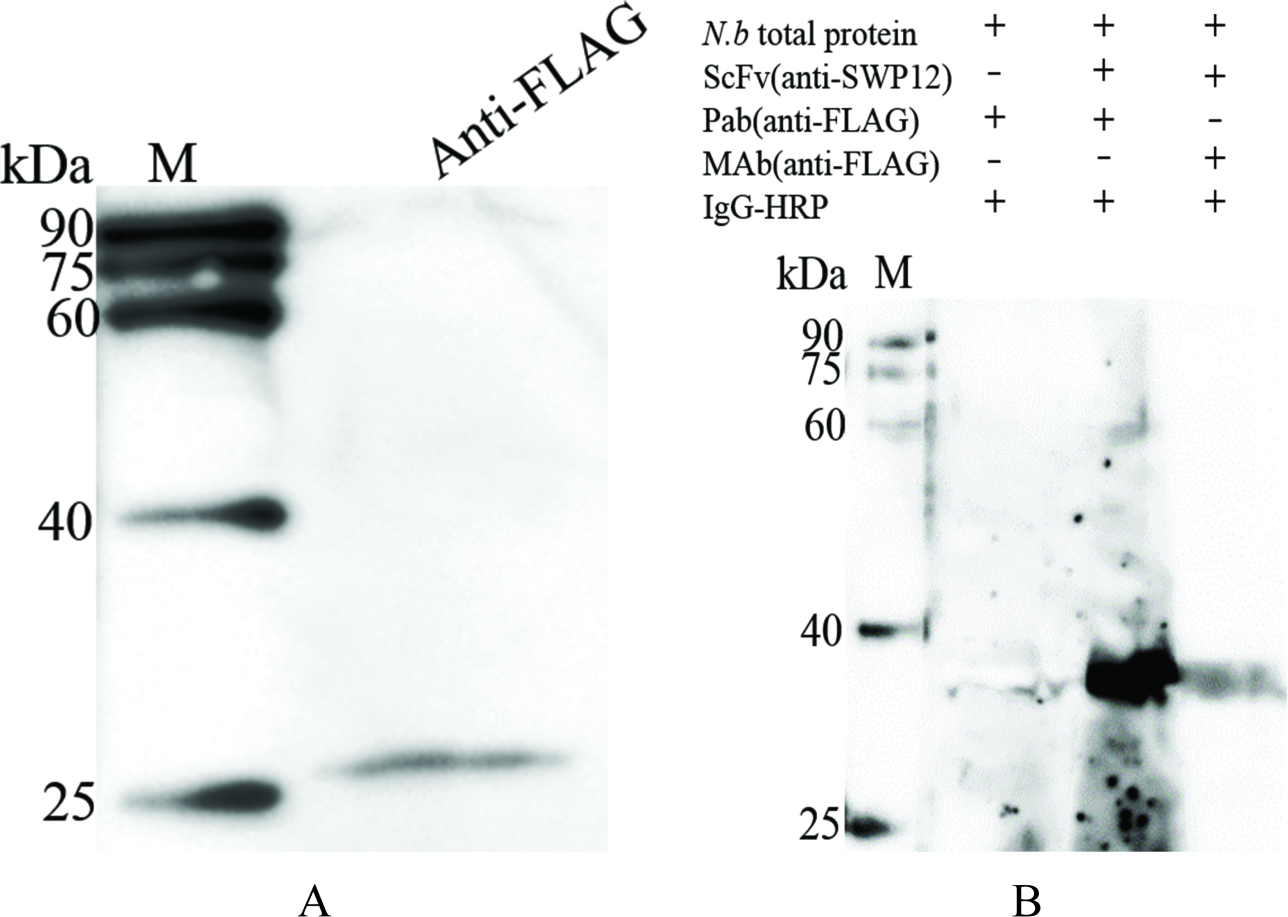
Analysis of scFv-G4 expression and its specificity. (A) Detection of scFv-G4 expression. Total protein from scFv transgenic Sf9-III cells was extracted and analyzed by SDS-PAGE. A FLAG mAb was used to detect the expression of scFv-G4. (B) Verification of scFv-G4 specificity. Spore protein were analyzed by SDS-PAGE. ScFv-G4 transgenic Sf9-III and negative control Sf9-III cell lysate solutions were used as the primary antibody, after which FLAG mAb or polyclonal antibodies were used as the secondary antibodies and then HRP-labeled goat IgG was used as the third antibody. M: protein marker (Transgene, China).

Western-blotting was conducted to detect if the scFv-G4 which was expressed in Sf9-III could target to SWP12 just like mAb G4. As scFv lacks the constant regions and cannot be recognized by anti-mouse or anti-rabbit IgG antibodies, a mouse anti-FLAG mAb (Sigma, Saint Louis, Missouri, USA) and a rabbit polyclonal antibody (Rockland Immunochemical, Philadelphia, Pennsylvania, USA) were used as secondary antibodies for scFv-G4 recognition in western blot. Recombinant scFv-G4 was expressed in Sf9-III cells and its successful expression was verified by western blotting with anti-FLAG antibody (Fig 7A). Western blotting also showed that scFv-G4 can specifically bind to native SWP12 (Fig 7B). The negative control (the second line of Fig 7B) illustrated the positive signal was not because of the nonspecific binding of the FLAG rabbit polyclonal antibody.

### *N*. *bombycis* challenge of transgenic and control Sf9-III

**Fig 8:**
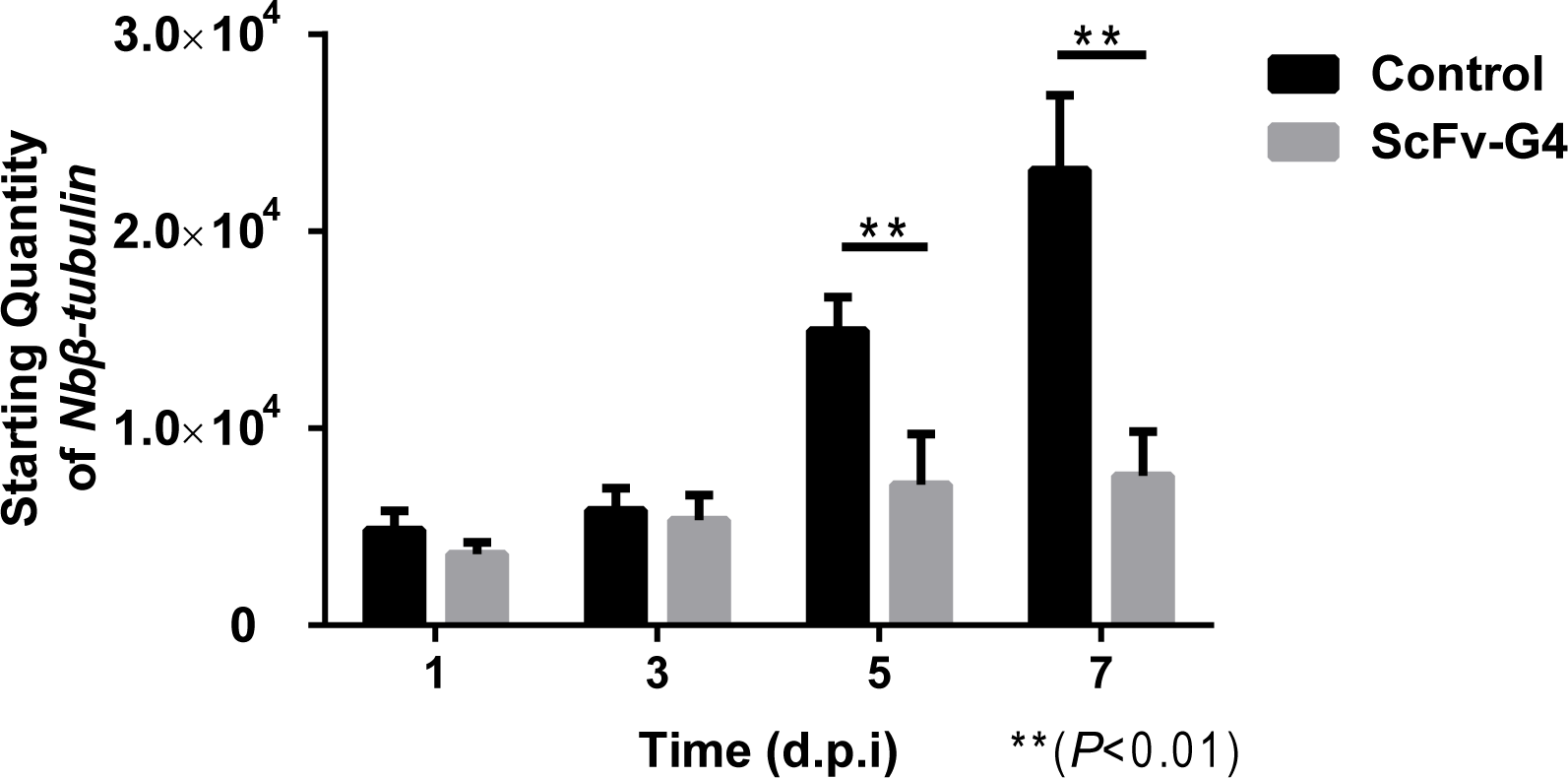
Effect of scFv-G4 expression on *N*. *bombycis* proliferation. Genomic DNA was extracted from infected scFv transgenic Sf9-III and control cells at 1, 3, 5, and 7 days post infection (d.p.i). Pathogen amounts were detected by Real-time quantitative PCR. Statistically significant differences are indicated with asterisks (*P*< 0.01). Bars represent the standard deviations of three independent replicates.

*β-tubulin* is a house keeping gene of *N*. *bombycis* which expressed consistently from the 1 d.p.i. [13]. Therefore, the copy number could reflect the number of *N*. *bombycis* and the Real-time quantitative PCR was conducted to detect the copy number of *N*. *bombycis β-tubulin* in infected cells. According to the Real-time quantitative PCR results, there were no significant proliferation differences between the control groups and experiment groups on d.p.i. 1 and 3. In the control groups, *N*. *bombycis* began to multiply at 3 to 7 d.p.i; however, *N*. *bombycis* numbers were markedly lower in the transgenic cells than in the control cells. Interestingly, the *N*. *bombycis* showed no signs of multiplication in transgenic cells, suggesting that the scFv-based anti-microsporidia strategy effectively inhibited their proliferation (Fig 8).

## Discussion

In previous research, the location of NbSWP12 in mature spores was confirmed by IFAs and transmission electron microscopy [13], but it was difficult to identify its location during the meront stage which lacks marker protein at that time. In our recent study, β-tubulin, the *N*. *bombycis* cytoskeletal protein on the inner side of the spore wall was proved to be a reliable meront stage marker protein, facilitating our further research on SWP12 [29]. In the IFAs, we failed to detected the SWP12 signal in the mature spore, which may cause by insufficient exposure of mAb G4 recognition epitope of SWP12. By using the polyclonal antibody which have multiple recognition epitopes, the signal of SWP12 on surface of mature spores was detected as expected (S4 Fig).

In research on yeast, the ability of BAR and F-BAR domain-containing proteins to induce tubular invaginations of the plasma membrane is enhanced by disruption of the actin cytoskeleton and is antagonized by dynamin [14]. According to the IFA results, NbSWP12 concentrated at both ends of the meronts, implying that SWP12 may play a particular role in stabilizing the plasma membrane during meront division. The combination of SWP12 and scFv-G4 interfered the SWP12 binding with its interacting protein. The meront membrane may become unstable without assist of SWP12 during its division, which would lead to *N*. *bombycis* bipartition failure. Furthermore, although *N*. *bombycis* contains many types of spore wall protein, perhaps not all the spore wall proteins are functional in the mature spores. Indeed, some spore wall proteins may also play roles in the meront stage.

In a previous study, Guo et al. expressed EGFP in *N*. *bombycis* via a non-transposon vector [30], but the transgenic *N*. *bombycis* was difficult to purify and continuous passage of the line was problematic. Therefore, a reliable gene editing method for gene function research in *N*. *bombycis* was lacking. To some extent, the scFv transgenic cell lines have offered a way to explore protein function in *N*. *bombycis*. However, it may be difficult to get scFv into the cytoplasm of *N*. *bombycis* in its natural state, so the use of scFv may be restricted to the study of surface and secretory proteins of *N*. *bombycis*.

In 2016, Gorawit Yusakul et al. expressed scFv (anti-paclitaxel) in *B*. *mori* [31]. Therefore, it is possible to express scFv-G4 in *B*. *mori* to protect it from *N*. *bombycis* infection, besides, we found that mAb G4 recognized SWP12 homolog proteins in *N*. *pernyi* and *N*. *papilio*, suggesting that this antibody may also function to protect *Antheraea pernyi* from these pathogens.

## Conclusion

*SWP12* not only express in mature stage of *N*. *bombycis*, but also in meront stage. Furthermore, SWP12 tended to be concentrated at both ends of the meronts, which imply it play an important role in division of *N*. *bombycis* during its meront stage. ScFv-G4 express in Sf9-III target to SWP12 can inhibit proliferation of *N*. *bombycis in vitro*. The outcome of our research offers a potential target against *N*. *bombycis* infection of *B*. *mori* and a strategy for breeding *N*. *bombycis*-resistant *B*. *mori*.

## Supporting information

S1 Fig Purification of rSWP12. Recombinant SWP12 was eluted by elution buffer contained different concentration imidazole. The SDS-PAGE showed rSWP12 was purified from recombinant *Escherichia coli* Rosetta by affinity chromatography, and the most of rSWP12 was eluted in elution buffer which contained 200mM imidazole.
(JPG)

S2 Fig Measurement of G4 titer. The G4 titer was detected by ELISA. Ascites of unimmunized BALB/c mouse and SP2/0 culture medium were as the negative control respectively. According to the ELISA results, (A) the titer of G4 hybridoma culture medium was 1:2048, while (B) the titer of ascites was 1:51200.
(JPG)

S3 Fig Constructed pSL-1180 and PiggyBac plasmid. IE1: Baculovirus *ie1* promoter; SV40: Simian virus 40 PolyA; A3: Promoter of *Bombyx mori actin 3*; pBacL/R: PiggyBac elements of transposition; Neo: Neomycin resistance gene; EGFP: Enhanced green fluorescent protein gene; Amp: Ampicillin resistance gene.
(TIF)

S4 Fig SWP12 subcellular localization in mature spores. Sf9-III cells were infected with *N*. *bombycis* and the resultant infections were visualized by confocal microscopy. Green fluorescence was observed in the samples treated with the polyclonal antibody against SWP12. The blue fluorescent signal represents the chitin coat labeled with FWA (Sigma, Saint Louis, Missouri, USA). The chitin layer blocked the binding of rabbit β-tubulin antiserum entry, so the red fluorescence was invisible.
(TIF)
