# Supplementary figures and images for: Engineered resistance to Nosema bombycis by in vitro expression of a single-chain antibody in Sf9-III cells
Source: PLoS One. 2018 Feb 15;13(2):e0193065. doi: 10.1371/journal.pone.0193065 (PMC5814085; doi:10.1371/journal.pone.0193065)

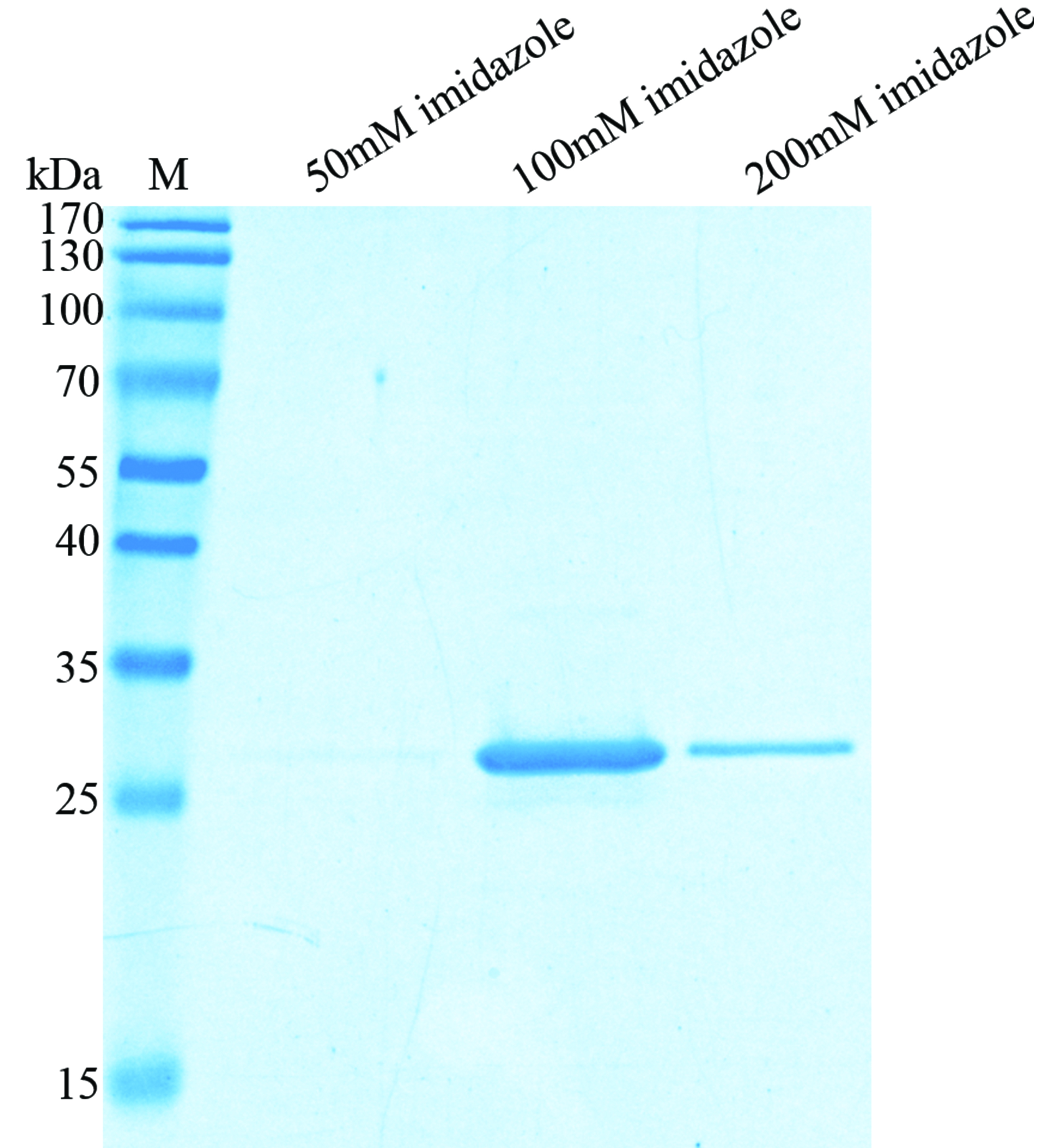

Supplement: S1 Fig — Recombinant SWP12 was eluted by elution buffer contained different concentration imidazole. The SDS-PAGE showed rSWP12 was purified from recombinant Escherichia coli Rosetta by affinity chromatography, and the most of rSWP12 was eluted in elution buffer which contained 200mM imidazole. (JPG) [file pone.0193065.s001.jpg]

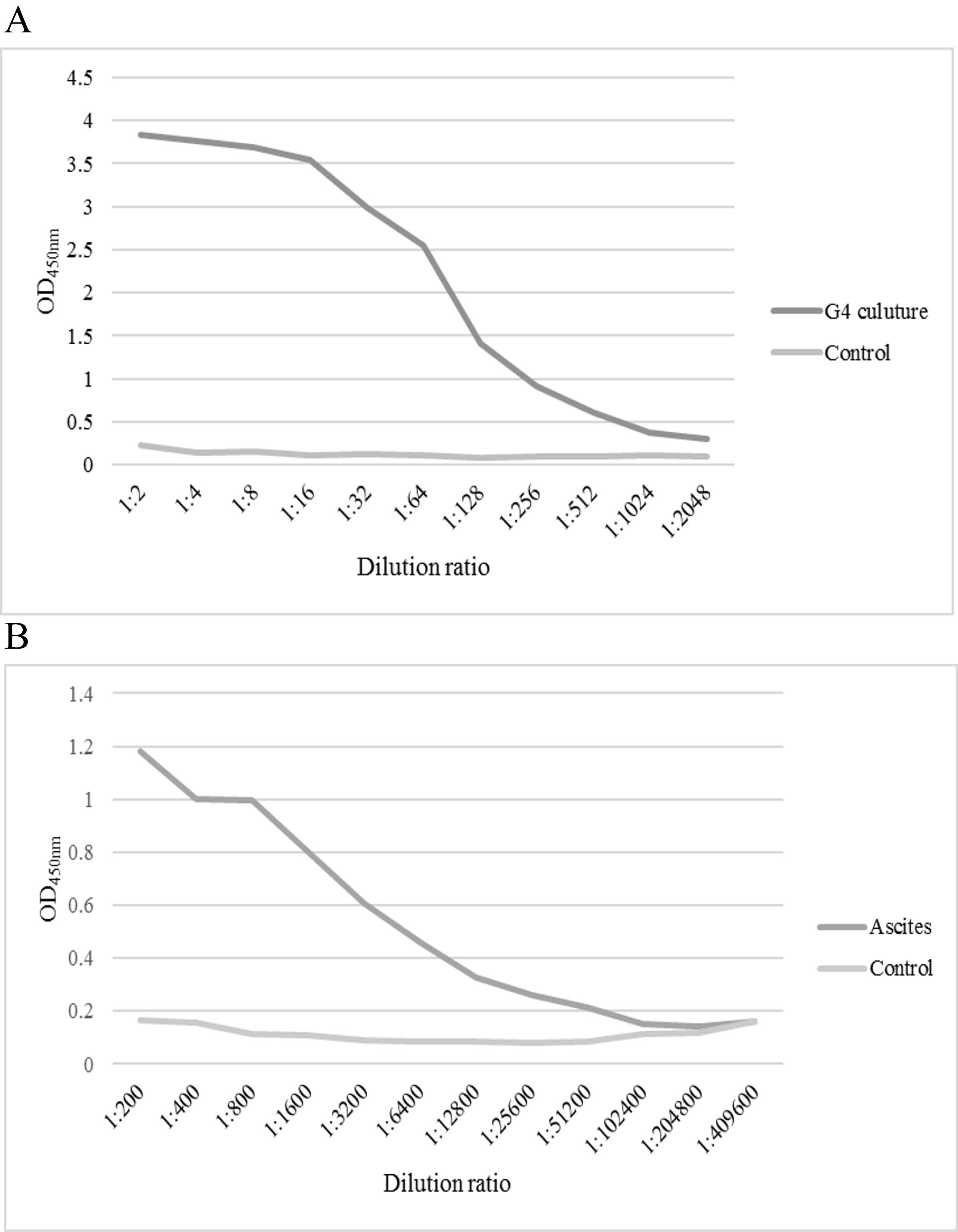

Supplement: S2 Fig — The G4 titer was detected by ELISA. Ascites of unimmunized BALB/c mouse and SP2/0 culture medium were as the negative control respectively. According to the ELISA results, (A) the titer of G4 hybridoma culture medium was 1:2048, while (B) the titer of ascites was 1:51200. (JPG) [file pone.0193065.s002.jpg]

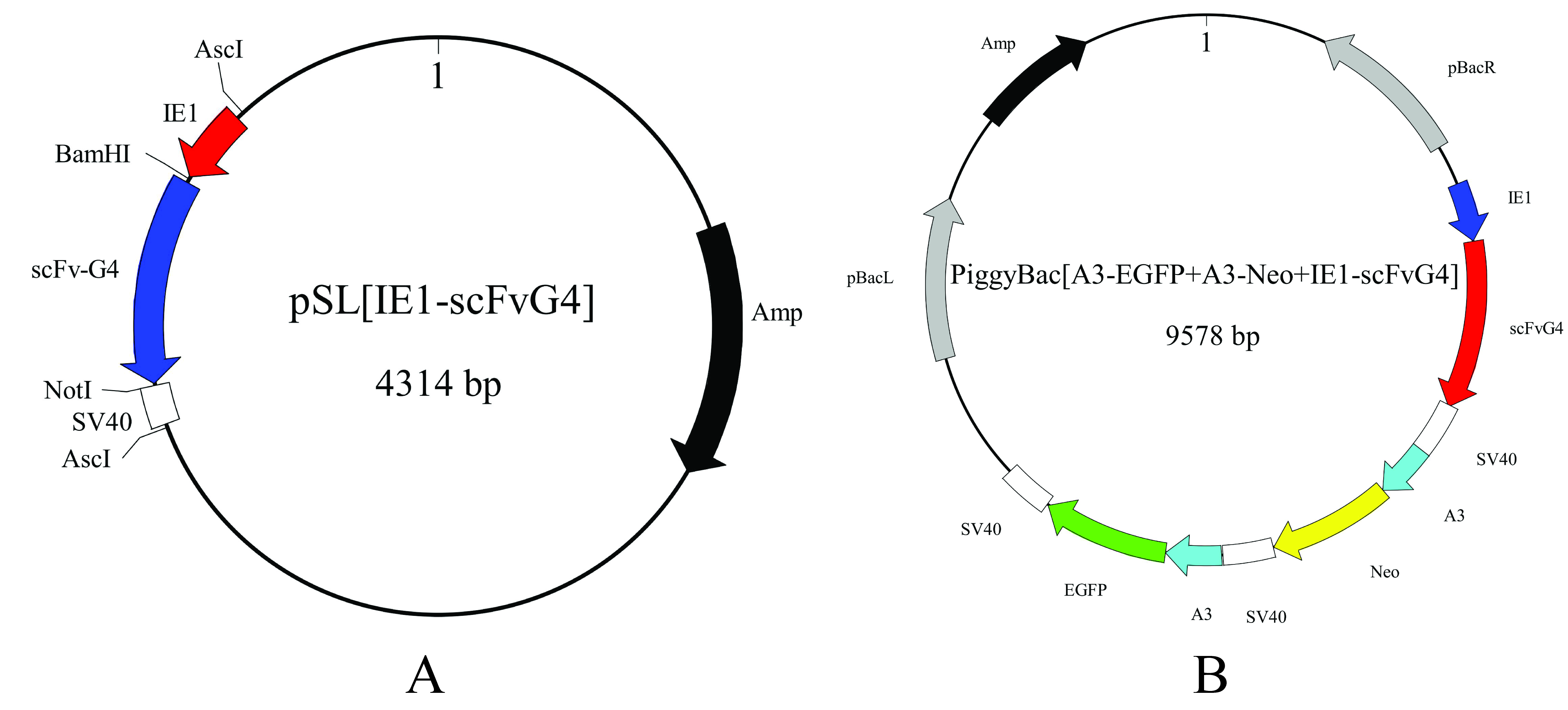

Supplement: S3 Fig — IE1: Baculovirus ie1 promoter; SV40: Simian virus 40 PolyA; A3: Promoter of Bombyx mori actin 3; pBacL/R: PiggyBac elements of transposition; Neo: Neomycin resistance gene; EGFP: Enhanced green fluorescent protein gene; Amp: Ampicillin resistance gene. (TIF) [file pone.0193065.s003.tif]

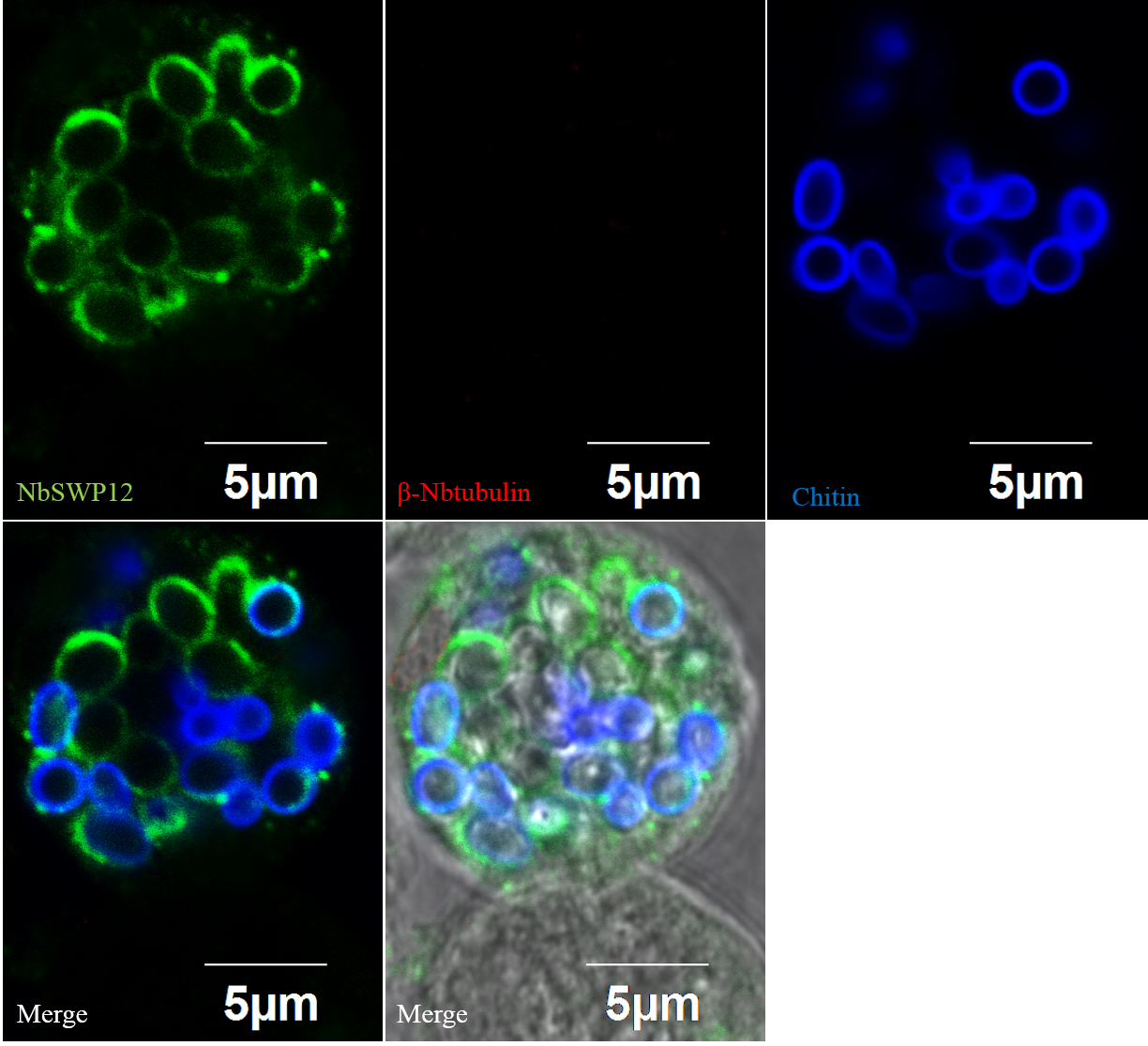

Supplement: S4 Fig — Sf9-III cells were infected with N. bombycis and the resultant infections were visualized by confocal microscopy. Green fluorescence was observed in the samples treated with the polyclonal antibody against SWP12. The blue fluorescent signal represents the chitin coat labeled with FWA (Sigma, Saint Louis, Missouri, USA). The chitin layer blocked the binding of rabbit β-tubulin antiserum entry, so the red fluorescence was invisible. (TIF) [file pone.0193065.s004.tif]
